# Exploring the Anticancer Potential of Traditional Thai Medicinal Plants: A Focus on *Dracaena loureiri* and Its Effects on Non-Small-Cell Lung Cancer

**DOI:** 10.3390/plants13020290

**Published:** 2024-01-18

**Authors:** Xiaomin Huang, Punnida Arjsri, Kamonwan Srisawad, Supachai Yodkeeree, Pornngarm Dejkriengkraikul

**Affiliations:** 1 Department of Biochemistry, Faculty of Medicine, Chiang Mai University, Chiang Mai 50200, Thailand; xiaomin_huang@cmu.ac.th (X.H.); punnida_a@cmu.ac.th (P.A.); kamonwan.sri@cmu.ac.th (K.S.); yodkeelee@hotmail.com (S.Y.); 2 Anticarcinogenesis and Apoptosis Research Cluster, Faculty of Medicine, Chiang Mai University, Chiang Mai 50200, Thailand; 3 Center for Research and Development of Natural Products for Health, Chiang Mai University, Chiang Mai 50200, Thailand

**Keywords:** *Dracaena loureirin*, non-small-cell lung cancer, anti-cancer properties, apoptosis

## Abstract

Non-small-cell lung cancer (NSCLC) is renowned for its aggressive and highly metastatic nature. In recent years, there has been a surge in interest regarding the therapeutic potential of traditional medicinal plants. *Dracaena loureirin* (*D. loureirin*), *Ficus racemosa* Linn. (*F. racemosa*), and *Harrisonia perforata* (Blanco) Merr. (*H. perforata*) are prominent traditional medicinal herbs in Thailand, recognized for their diverse biological activities, including antipyretic and anti-inflammatory effects. However, their prospective anti-cancer properties against NSCLC remain largely unexplored. This study aimed to evaluate the anti-cancer attributes of ethanolic extracts obtained from *D. loureiri* (DLEE), *F. racemosa* (FREE), and *H. perforata* (HPEE) against the A549 lung adenocarcinoma cell lines. Sulforhodamine B (SRB) assay results revealed that only DLEE exhibited cytotoxic effects on A549 cells, whereas FREE and HPEE showed no such cytotoxicity. To elucidate the anti-cancer mechanisms of DLEE, cell cycle and apoptosis assays were performed. The findings demonstrated that DLEE inhibited cell proliferation and induced cell cycle arrest at the G0/G1 phase in A549 cells through the downregulation of key cell cycle regulator proteins, including cyclin D1, CDK-2, and CDK-4. Furthermore, DLEE treatment facilitated apoptosis in A549 cells by suppressing anti-apoptotic proteins (Bcl-2, Bcl-xl, and survivin) and enhancing apoptotic proteins (cleaved-caspase-3 and cleaved-PARP-1). In summary, our study provides novel insights into the significant anti-cancer properties of DLEE against A549 cells. This work represents the first report suggesting that DLEE has the capability to impede the growth of A549 lung adenocarcinoma cells through the induction of apoptosis.

## 1. Introduction

Lung cancer stands as the foremost cause of cancer-related fatalities globally [1]. It can be classified into two types: small-cell lung carcinoma (SCLC) and non-small-cell lung carcinoma (NSCLC), with the latter representing about 85% of lung cancer cases. The main etiological factors contributing to NSCLC include smoking or second-hand smoke, exposure to radon gas and other toxic substances, as well as genetic factors. Although patients with NSCLC in the early stages can achieve complete recovery through surgery, approximately half of those who undergo the surgical procedure experience a relapse, and detecting it early becomes challenging. Consequently, individuals diagnosed with NSCLC have lower five-year survival rates [2]. When considering the alternative therapeutic strategy of combining surgery with chemotherapeutic drugs for lung cancer, it is important to acknowledge that many patients experience adverse effects, including chemoresistance, recurrence, and damage to normal tissue [3,4]. Cisplatin (cis-diamminedichloroplatinum(II), CDDP) is commonly used as a chemotherapeutic drug for various types of tumors, including lung, ovarian, breast, and testicular cancers [5,6]. However, CDDP treatment often leads to side effects such as nausea, vomiting, visceral toxicity, and tumor resistance. Therefore, it is crucial to discover more effective compounds with fewer side effects for aggressive cancers like lung cancer. In recent decades, numerous natural compounds, such as paclitaxel, vinblastine, camptothecin, and oleuropein, have been identified and extensively employed as anti-cancer agents [7]. Natural products offer a reliable source for drug discovery.

Folk medicine has been introduced as adjuvant therapy to delay cancer growth and development despite the lack of scientific evidence. Thus, it should be noted that many medicinal plants require more research data to support their application in folk medicine. Phytocompounds play a significant role in cancer prevention and the treatment of cancer. Secondary metabolites, known for their anti-inflammatory, antioxidant, and anticancer properties, are extensively studied for the discovery of new therapeutic compounds [8,9]. Numerous medicinal plants in Thailand are recognized for their medicinal properties. *Ficus racemosa*, *Harrisonia perforata*, and *Dracaena loureiri* are examples of plants rich in polyphenols that can be utilized in cancer treatment.

*Ficus racemosa* Linn., a member of the Moraceae family, is native to Southeast Asia and India and has been utilized as an herbal remedy for centuries. Commonly known as “gular” or “Dumur”, this plant’s leaves have been traditionally used to treat various ailments, including piles, dysentery, diarrhea, and skin conditions. They are also recognized for their anthelmintic, astringent, antidiabetic, and anti-inflammatory properties [10,11,12].

*Harrisonia perforata* (Blanco) Merr., a member of the Harrisonia genus of the Simarubaceae family and is locally known as “Khon-Tha” in Thailand. This prickly shrub is widely distributed throughout Southeast Asia. Different plant parts, including the roots, stems, leaves, and branches, have been employed in traditional medicine for addressing conditions such as fever, diarrhea, dysentery, malaria, wound healing, and cancer [13,14,15]. Previous phytochemical studies have identified limonoids, polyketides, and chromones as the main chemical constituents of this plant, exhibiting fascinating bioactivities such as cytotoxicity [13], anti-inflammatory effects [16], anti-tobacco mosaic virus activity [17], and anti-mycobacterial and anti-plasmodial activities [18].

*Dracaena loureiri* Gagnep is a medicinal plant that is widely cultivated in China and Southeast Asia. It can be found in various parts of Thailand and is known as “Jun-Daeng” or “Thai Dracaena” among the locals. In folk medicine, this herb has been traditionally used to promote blood circulation, facilitate tissue regeneration for wound healing, relieve pain, and reduce swelling. It has been commonly employed in the treatment of conditions such as coronary heart diseases, angina, acute myocardial infarction, antimalarial effects, antiulcer properties, antidiarrheal effects, and antidiabetic activity [19,20,21]. Previous studies have identified potential phenolics and flavonoids as the chemical constituents of *D. loureiri*. These include loureirins A–D, loureiriol, and stilbenoid derivatives [19,22]. These active constituents have demonstrated potential biological activities such as anti-inflammatory effects through the regulation of the NF-κB signaling pathway and anti-cancer properties [23]. However, the specific anti-cancer effects of this plant on lung cancer progression have not yet been elucidated.

However, no published data currently exist regarding the anti-cancer properties of these three medicinal herbs specifically in non-small-cell lung cancer cell phenotypes. This lack of information has motivated us to explore the anti-cancer potential of these medicinal herbs, considering their previously reported potent anti-inflammatory activities. The objectives of this study were to investigate the potential anti-cancer properties of three Asian medicinal herbal extracts on lung cancer and assess the underlying mechanisms by which these herbal extracts inhibit cell proliferation and promote apoptosis in lung cancer cells.

## 2. Results

### 2.1. The Total Phenolic and Total Flavonoid Contents in DLEE, HPEE, and FREE

After completing the ethanolic extraction process, three distinct ethanolic extracts were obtained: *Dracaena loureiri* ethanolic extract (DLEE) with a yield of 16.00%, *Harrisonia perforata* ethanolic extract (HPEE) with a yield of 6.05%, and *Ficus racemosa* ethanolic extract (FREE) with a yield of 5.78%. The total phenolic contents of three ethanolic extracts were quantified by Folin–Ciocalteu assay. The results revealed that DLEE exhibited the highest phenolic content (251.49 ± 23.13 mg of GAE/g of extract), followed by HPEE (193.53 ± 3.21 mg of GAE/g of extract), and FREE (89.05 ± 3.42 mg of GAE/g of extract) significantly (*p* < 0.001). Moreover, DLEE demonstrated the highest total flavonoid content (85.83 ± 4.80 mg of CE/g of extract), followed by HPEE (62.13 ± 1.22 mg of CE/g of extract) and FREE (46.64 ± 1.59 mg of CE/g of extract), respectively as shown in Table 1. From the results, it can be concluded that the ethanolic extracts of these three plants contain a high amount of phenolic and flavonoid compounds and that these extracts can be used for investigation of their anti-cancer properties in further experiments.

### 2.2. HPLC Analysis of Phytochemical Constituents in DLEE

Due to the substantial levels of total phenolic and total flavonoid contents found in DLEE, we proceeded to identify its phytochemical constituents using the HPLC technique. Standard compounds, such as loureirin A, loureirin B, resveratrol, rutin, quercetin, quercitrin, catechin, luteolin, apigenin, hesperetin, and hesperidin were employed for the identification of the phytochemical constituents present in DLEE. The results indicate loureirin A and quercetin as the predominant compounds in DLEE, present at concentrations of 28.11 ± 0.34 and 25.81 ± 1.12 mg/g of extract, respectively. Additionally, significant amounts of quercitrin, loureirin B, hesperetin, rutin, catechin resveratrol, and hesperidin were also identified, as detailed in Table 2. This HPLC analysis revealed multiple phytochemical constituents within DLEE, suggesting their potential significance in contributing to the biological activity of the extract.

### 2.3. Evaluation of the Anti-Cancer Potential of DLEE, HPEE, and FREE against A549 Cells

**Figure 1:**
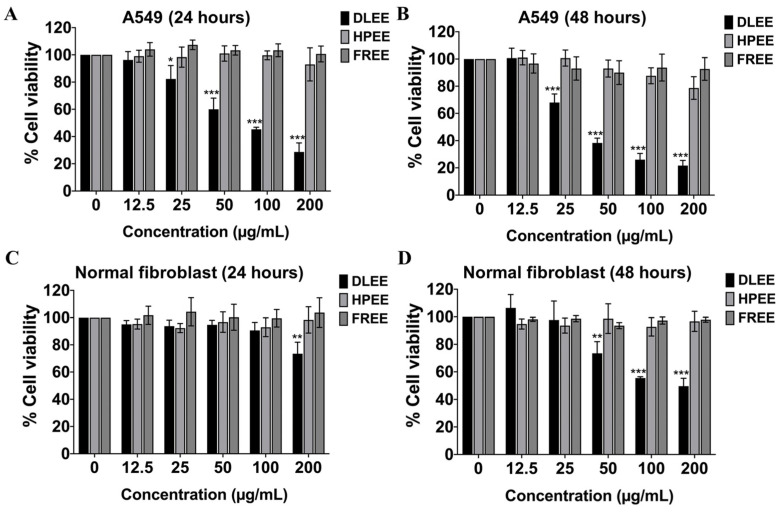
The effects of DLEE, HPEE, or FREE on A549 (**A**,**B**) and human normal fibroblast (**C**,**D**) cell viability were determined using the SRB assay. A549 cells and human normal fibroblast cells were treated with DLEE, HPEE, or FREE at concentrations ranging from 0 to 200 µg/mL for 24 h and 48 h. The data are expressed as mean ± S.D. values from three independent experiments. * *p* < 0.05, ** *p* < 0.01, and *** *p* < 0.001 compared with the control.

To evaluate the anti-cancer properties of these ethanolic extracts (DLEE, HPEE, and FREE), the cell viability of A549 cells was determined by the SRB assay. The results revealed that, among the diverse herbal extracts, DLEE exhibited the most remarkable inhibitory impact on the growth of A549 cells. This pronounced effect was evident by an IC_50_ (50% inhibitory concentration) value of 76.25 ± 4.53 μg/mL for 24 h and 38.45 ± 3.48 μg/mL for 48 h of DLEE treatment, as shown in Figure 1A,B. Conversely, both HPEE and FREE exhibited negligible cytotoxic effects on A549 cells, as displayed in Figure 1A, respectively. These findings unequivocally highlight the potent anti-cancer attributes of DLEE, whereas HPEE and FREE exhibit no anti-cancer properties.

Phytochemicals or natural compounds are well known for their eco-friendly and safe properties. However, we also demonstrated the cytotoxicity of DLEE, HPEE, and FREE on a human normal fibroblast cell line serving as the control for normal cells. The results revealed that after 24 h of incubation, DLEE, HPEE, and FREE exhibited no cytotoxic effects on human fibroblast cells. However, after 48 h, DLEE demonstrated cytotoxicity with an IC_50_ of 200.33 ± 6.81 µg/mL, while HPEE and FREE continued to show no cytotoxicity, as is displayed in Figure 1C,D. The selectivity index (SI) of DLEE on A549 cells and human fibroblast cells was calculated as 5.21 at 48 h of incubation. Meanwhile, DLEE maintained a selectivity index exceeding five for A549 cells, indicating its preferential impact on these cells, while exhibiting no adverse effects on normal cells at the concentrations employed in our experimental studies.

### 2.4. Reduction of Colony Formation by DLEE in A549 Cells

**Figure 2:**
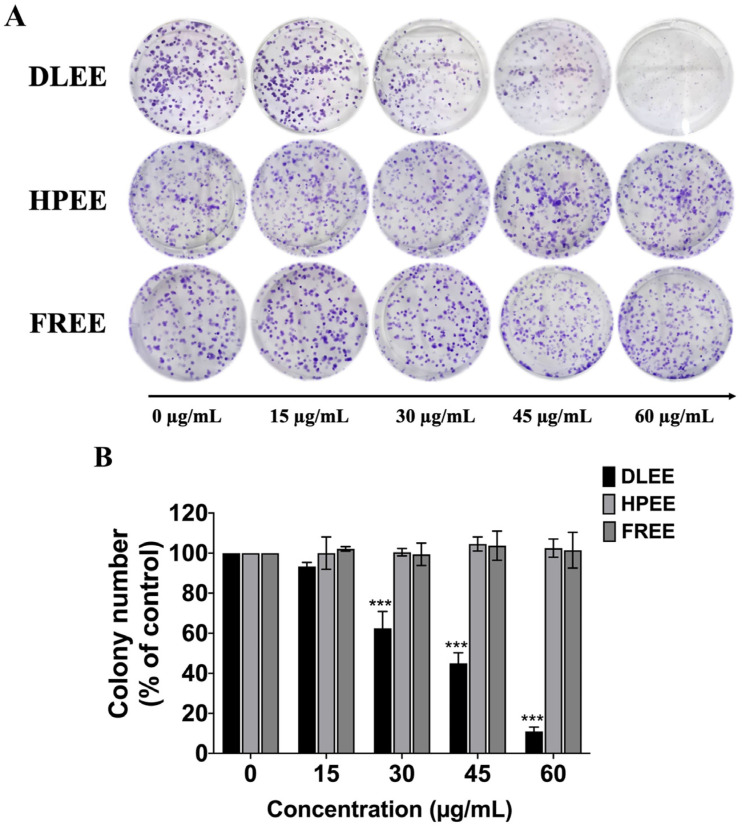
Impact of DLEE, HPEE, and FERR on A549 cell proliferation using a colony formation assay. The A549 cells were exposed to varying concentrations (0–60 µg/mL) of DLEE, HPEE, or FERR for a duration of 7 days. The former colonies were photographed using phase-contrast microscopy (**A**) and quantified using Image J 1.410 software (**B**). The data are expressed as mean ± S.D. values from three independent experiments. *** *p* < 0.001 compared to the control group.

To investigate the anti-proliferative properties of DLEE, HPEE, and FREE against A549 cells, and building upon the findings presented in Figure 1, where only DLEE exhibited discernible anti-cancer properties in comparison to the other two plants (HPEE and FREE), we sought to further confirm these anti-cancer properties through a colony formation assay. This assay is commonly employed to determine the potential of single-cell proliferation by observing colony formation, providing insights into the drug sensitivity of cancer cells. To assess the effect of DLEE on A549 cell proliferation, the colony formation assay was determined. The results demonstrated a significant and dose-dependent reduction in colony formation with DLEE treatment (*p* < 0.001). Conversely, the two other herbal extracts exhibited no discernible anti-proliferative effects on A549 cells (Figure 2A,B). Overall, our findings conclusively indicate that DLEE possesses remarkable anti-cancer properties against A549 cells, manifested through the inhibition of A549 cell proliferation.

### 2.5. Effect of DLEE on Cell Cycle Arrest in A549 Cells

**Figure 3:**
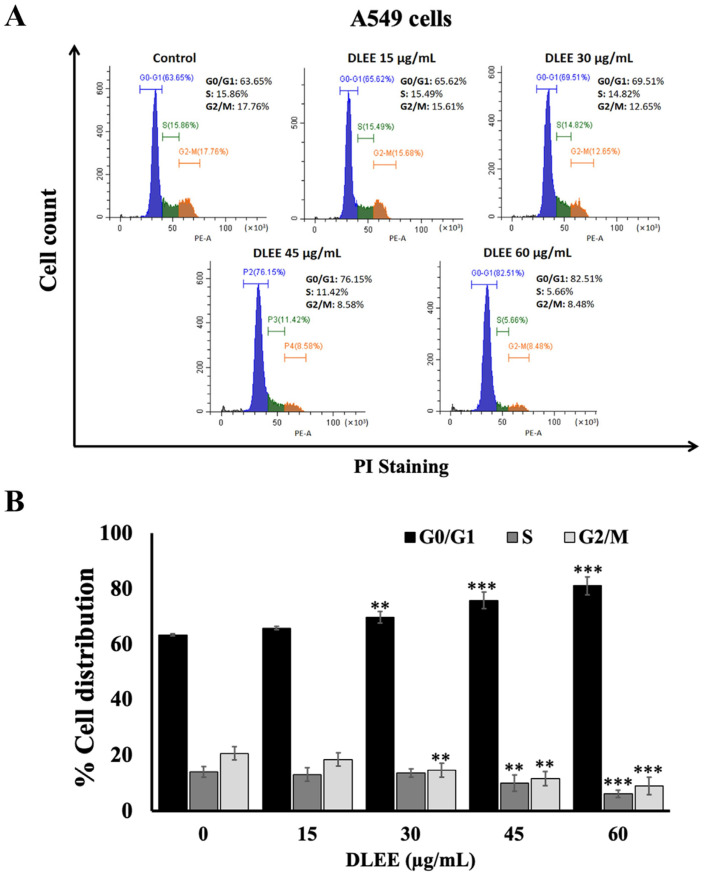
Impact of DLEE on A549 cell cycle distribution. The A549 were treated with DLEE at concentrations ranging from 0 to 60 µg/mL cells for 24 h. The cells were stained with propidium iodide (PI) before analysis by flow cytometer (**A**). The graphs show the populations of A549 cells in the G0, S, and G2/M phases (**B**). The data are expressed as mean ± S.D. values from three independent experiments. ** *p* < 0.01 and *** *p* < 0.001 compared to the control.

Based on the data obtained from both the SRB and colony formation assays, DLEE was selected to further investigate the underlying mechanisms associated with anti-cancer properties. To assess the influence of DLEE on A549 cell proliferation, the cell distribution of A549 cells in various cell cycle phases was analyzed by staining with propidium iodide (PI) and analyzed by flow cytometer. Subsequent to treatment with DLEE, a substantial dose-dependent increase was observed in the population of A549 cells residing in the G0/G1 phase (*p* < 0.001). Simultaneously, a pronounced dose-dependent reduction was evident in the population of cells in the S and the G2/M phases (*p* < 0.001), as illustrated in Figure 3A,B. These findings collectively affirm that DLEE induced A549 cell cycle arrest specifically at the G0/G1 phase.

### 2.6. Effects of DLEE on Proteins Involved in G0/G1 Phase Arrest in A549 Cells

**Figure 4:**
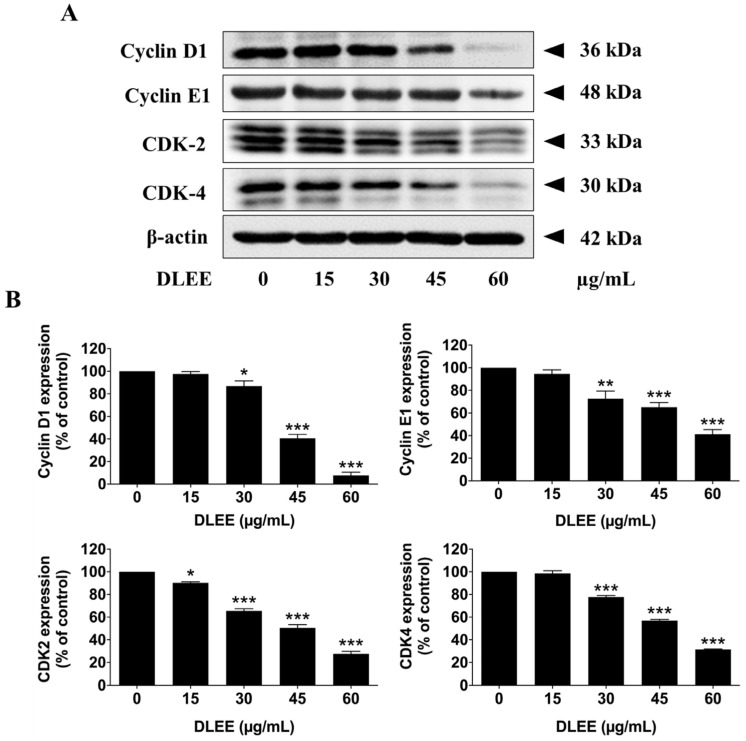
The effects of DLEE on the expression of cell cycle-regulated proteins (Cyclin D1, Cyclin E1, CDK-2, and CDK-4). The A549 cells were treated with various concentrations of DLEE, ranging from 0 to 60 µg/mL, for 24 h before harvesting. Protein expression was assessed using the Western blot technique (**A**), and the density of the band was quantified using Image J 1.410 software (**B**). The data are expressed as mean ± S.D. values from three independent experiments. * *p* < 0.05, ** *p* < 0.01, and *** *p* < 0.001 compared to the control.

Considering the pivotal role of the G1 phase in the cell cycle progression of A549 cells, where cells prepare for entry into the S phase through the regulation of cyclin D1, cyclin E1, CDK-2, and CDK-4, it becomes apparent that the formation of cyclins/CDKs complexes regulate DNA synthesis in preparation for cell division [24]. To elucidate the mechanistic actions of DLEE, which regulates cell cycle arrest in A549 cells, the expression levels of key cell cycle regulatory proteins after treatment with DLEE were investigated by Western blotting analysis. The results of Western blot analysis revealed a significant dose-dependent reduction in the expression levels of cyclin D1, cyclin E1, CDK-4, and CDK-2 following DLEE treatments (*p* < 0.001), as illustrated in Figure 4A,B. These findings underscore the proficiency of DLEE in inducing cell cycle arrest and impeding cell proliferation in A549 cells at the G0/G1 phase, primarily attributed to the suppression of the expression of the cyclin D1, cyclin E1, CDK-2, and CDK-4 proteins.

### 2.7. Evaluation of DLEE’s Impact on Apoptosis in A549 Cells

**Figure 5:**
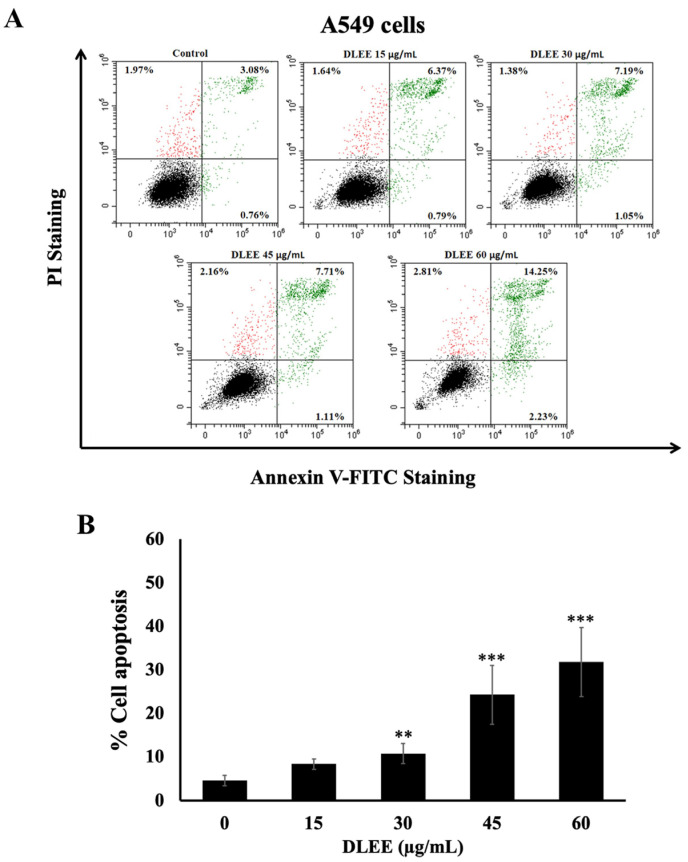
The effect of DLEE on the apoptosis of A549 cells. The A549 cells were treated with various concentrations of DLEE ranging from 0 to 60 µg/mL for 48 h before co-staining with PI and Annexin V-FITC dyes. Cell death was analyzed using flow cytometer (**A**), and the graphs plot the % of cell apoptosis (**B**). The data are expressed as mean ± S.D. values from three independent experiments. ** *p* < 0.01 and *** *p* < 0.001 compared to the control.

In the context of this investigation, aimed at substantiating that the cytotoxic mechanism of DLEE in A549 cells is attributed to the induction of apoptosis, A549 cells were subjected to varying concentrations of DLEE (0, 15, 30, 45, and 60 μg/mL) for a duration of 48 h. Apoptosis induction was subsequently assessed using Annexin V-FITC and PI staining, and the results were analyzed through flow cytometry. The results demonstrate that DLEE treatment significantly increased apoptotic cell populations in a dose-dependent manner (*p* < 0.001), as illustrated in Figure 5A,B. These observations provide compelling substantiation for the capacity of DLEE to induce apoptosis in A549 cells.

### 2.8. Impact of DLEE on Pro- and Anti-Apoptotic Protein Expression in A549 Cells

**Figure 6:**
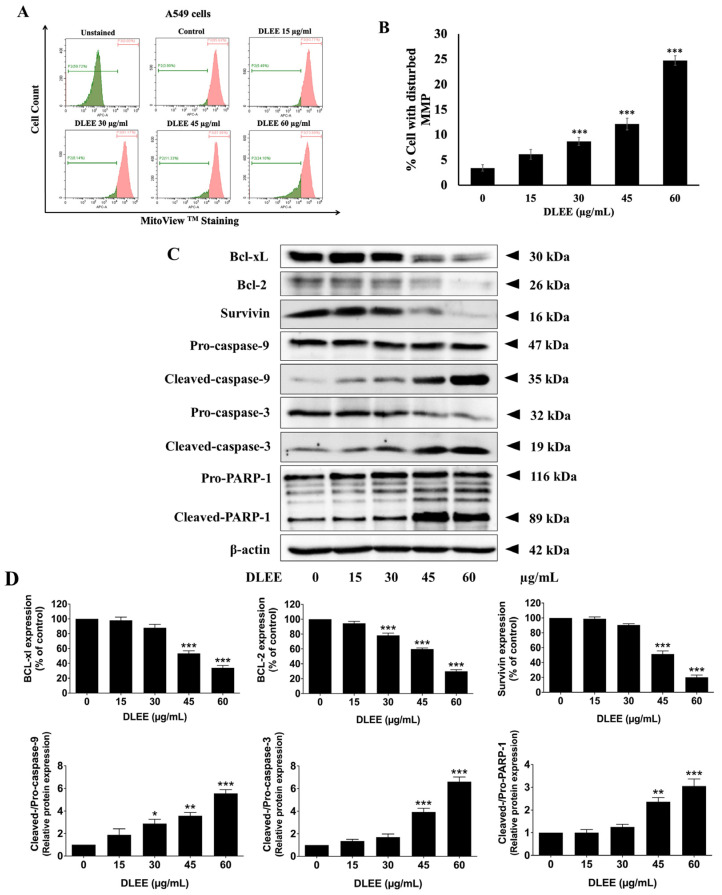
Effects of DLEE to induce intrinsic apoptosis cascade in A549 cells. The A549 cells were treated with various concentrations of DLEE ranging from 0 to 60 µg/mL for 48 h before harvesting. Following treatment, the mitochondrial membrane potential (MMP(ΔΨm)) of A549 cells was assessed by staining with MitoViewTM dye (**A**,**B**). The expression of anti-apoptotic proteins (survivin, Bcl-2, and Bcl-xl) and the activation of pro-apoptotic proteins (cleaved-caspase-9, cleaved-caspase-3, and cleaved-PARP-1) in A549 cells were analyzed by Western blot (**C**). The density of the band was quantified using Image J 1.410 software (**D**). The data are expressed as mean ± S.D. values from three independent experiments. * *p* < 0.05, ** *p* < 0.01, and *** *p* < 0.001 compared to the control.

To gain deeper insights into the molecular mechanisms underlying the induction of apoptosis by DLEE in A549 cells, the mitochondrial membrane potential (MMP, ΔΨm) alterations were assessed through MitoView^TM^ dye staining. DLEE-treated A549 cells exhibited a significant increase in the percentage of cells with disturbed mitochondrial membrane potential compared to the control (*p* < 0.001) (Figure 6A,B). The observed decrease in ΔΨm triggered the activation of the intrinsic apoptosis signaling cascade. Subsequently, we investigated the expression levels of key apoptosis-related proteins, including Bcl-xl, Bcl-2, surviving, cleaved-caspase-9, cleaved-caspase-3, and cleaved-PARP-1, using Western blot analysis. The results indicated a dose-dependent downregulation of Bcl-xl, Bcl-2, and survivin expression, accompanied by an increase in the activation of cleaved-caspases-9, cleaved-caspases-3, and cleaved-PARP-1 upon DLEE treatment (*p* < 0.001) in A549 cells (Figure 6C,D). As depicted in Figure 6, our findings present compelling evidence supporting the assertion that DLEE induces apoptosis in A549 cells via the activation of the intrinsic apoptosis pathway.

## 3. Discussion

Medicinal plants have played a pivotal role in traditional medicine for decades, offering favorable therapeutic effects with minimal toxicity [25,26]. Numerous studies have reported the diverse biological activities of medicinal herbs, including their anti-cancer properties, such as anti-proliferation, apoptosis induction, anti-metastasis, and anti-angiogenesis [27]. Numerous phytocompounds present in medicinal herbs have undergone extensive investigation to elucidate their molecular targets, aiming to establish an alternative strategy for targeted cancer treatments [9,28]. Nonetheless, in folk or Ayurveda medicine, medicinal plants are traditionally harvested and subjected to solute diffusion with ethanol for a defined period before storage, prior to their eventual application [29]. Following Ayurvedic herbal drug processing, to improve the mobility of the phytochemical compounds, characterized as medium-polarity molecules, from the medicinal plants to the solvent and enhance cell permeability, 80% ethanol was used for the extraction of three medicinal plants in our study.

In this study, we explored the anti-cancer potential of three traditional medicinal herbs, *Dracaena loureiri*, *Harrisonia perforata*, and *Ficus racemosa*, which are known for their rich content of phenolic and flavonoid phytochemicals [12,15,19]. Our investigation revealed that DLEE exhibited significant cytotoxic effects on A549 cells, distinguishing it from FREE and HPEE. This led us to focus our subsequent analyses on DLEE, emphasizing its potential in cancer treatments. We employed SRB and colony formation assays, which indicated that DLEE exerted anti-cancer effects on A549 cells.

Cell cycle analysis revealed that DLEE induced G0/G1-phase cell cycle arrest in A549 cells, an effect associated with the downregulation of key cell cycle-regulated proteins, including cyclin D1, cyclin E1, CDK-2, and CDK-4 [30]. The cyclins/CDKs complexes such as cyclin D1/CDK-4 and cyclin E1/CDK-2 complex were required to start the DNA replication process [24]. However, further mechanistic insights into the specific pathways affected by DLEE in inducing cell cycle arrest merit exploration.

Cell cycle arrest is associated with the triggering of cell death and usually occurs during the induction of tumor cell apoptosis [31,32]. Apoptosis, also known as programmed cell death, is a cellular suicide process governed by intracellular mechanisms [33]. The intrinsic pathway also referred to as the mitochondrial pathway, is triggered by intracellular signals initiated when the mitochondrial membrane undergoes a loss of potential (ΔΨm). Subsequently, cytochrome c is liberated from the mitochondrial intermembrane space into the cytosol, activating a cascade of caspase enzymes that ultimately culminate in apoptosis [34,35]. The subsequent investigation into apoptosis mechanisms demonstrated that DLEE induced apoptosis in A549 cells through the intrinsic pathway, as evidenced by the disruption of mitochondria membrane potential (ΔΨm) and activation of pro-apoptosis proteins, including cleaved-caspase-9, cleaved-caspase-3, and cleaved-PARP-1, while inhibiting anti-apoptotic proteins such as Bcl-2, Bcl-xl, and survivin. Previously recognized for various bioactivities, such as anti-malarial, anti-ulcer, anti-diabetic, and anti-inflammatory effects, *D. loureirin* has demonstrated high antioxidant activity [19,21,36,37,38]. However, this study represents the first report of *D. loureiri* extract exhibiting anti-cancer activities against non-small-cell lung cancer through the inhibition of cell proliferation and induction of apoptosis. It is crucial to delve deeper into the signaling cascades involved in the intrinsic pathway to understand the molecular events leading to apoptosis.

A previous investigation indicated that the ethanolic extract of *D. loureiri* boasts a substantial concentration of pharmaceutically important phytochemicals, notably belonging to the flavonoid and stilbenoid groups such as loureirins A-D, dihydroxyflavone, quercetin, trans- and cis-stilbene, resveratrol, and rutin [19,20,39,40,41]. A previous study found that loureirin A could downregulate the Akt/NF-κB signaling pathway in chondrocytes in vitro and in vivo studies [42]. The quercetin compound exhibited the anti-cancer effect against human lung cancer cells by apoptosis induction [43]. Resveratrol has been reported to possess apoptosis-inducing properties in different human cancer cell types, including breast (MCF-7 cells), liver (HepG2 cells), and lung (A549 cells) cancer cells [44,45]. Trans- and cis-stilbene polyphenols, have demonstrated their capability to induce rapid perinuclear mitochondrial clustering and p53-independent apoptosis in HCT116 colorectal carcinoma cells but not in normal cells [46]. Rutin has been documented to enhance A549 human lung carcinoma cell apoptosis through the induction of the TNF-α signaling pathway [47]. Additionally, a previous study has explored the anti-cancer properties of hesperetin on non-small-cell lung cancer (NSCLC) cells [48,49]. In our investigation, HPLC analysis indicated the multiple phytochemical constituents within DLEE, such as loureirin A, quercetin, quercitrin, loureirin B, hesperetin, rutin, catechin resveratrol, and hesperidin, suggesting their potential significance in contributing to the biological activity of the extract. These findings underscore the importance of exploring the individual contributions of these phytochemicals to the observed anti-cancer efficacy. Therefore, the data presented in our research provide scientific evidence that *D. loureiri* ethanolic extract displays antiproliferative and anticancer effects by growth inhibition and apoptosis induction.

Moreover, a comparative analysis with other known anti-cancer agents or treatments for non-small-cell lung cancer could provide insights into the uniqueness and potential advantages of *D. loureirin* extract. Additionally, addressing potential limitations, such as dose-dependent effects and resistance mechanisms, is crucial for the translational potential of DLEE. While our in vitro findings demonstrate the anticancer potential of DLEE, further validation through in vivo experiments is essential. Future investigation should focus on assessing the pharmacokinetics, systemic effects, and safety profile of DLEE in relevant animal models. Additionally, exploring potential synergies with existing therapies and addressing dose-dependent effects will contribute to the translational potential of DLEE in non-small-cell lung cancer treatment. Looking ahead, future research could explore the synergistic effects of DLEE with existing chemotherapeutic agents or investigate its efficacy in combination with immunotherapy for non-small-cell lung cancer. Moreover, the safety profile of *D. loureirin* extract, including potential toxic effects, should be considered for a comprehensive evaluation of its therapeutic potential.

In conclusion, our study presents compelling evidence supporting the anti-cancer properties of DLEE. This is achieved through the regulation of cell cycle-regulated proteins, leading to cell cycle arrest at the G0/G1 phase, and induction of apoptosis by modulating the expression of pro- and anti-apoptotic proteins. Therefore, *D. loureirin* could be a potential medicinal plant for non-small-cell lung cancer treatment, offering a unique combination of phytochemicals with anti-cancer efficacy. These findings contribute to advancements in drug discovery and development, paving the way for improved management of non-small-cell lung cancer.

## 4. Materials and Methods

### 4.1. Herb Materials

The commercially available herb powders including *Dracaena loureiri, Harrisonia perforata*, and *Ficus racemosa* were obtained from Buan-hua-tueng herbs drug store in Phisanulok, Thailand (Thai traditional medicine license 27922). The voucher specimen numbers of *Dracaena loureiri* (No. Wd053), *Harrisonia perforatea* (No. RT046), *Ficus racemosa* (No. RT048), were certified at the herbarium of the Flora of Thailand, Chiang Mai University, Thailand.

### 4.2. Reagents and Chemicals

Dulbecco’s modified eagle medium (DMEM), fetal bovine serum (FBS), 2.5% trypsin, and penicillin-streptomycin were obtained from Gibco BRL Company (Grand Island, NY, USA). The protease inhibitor cocktail, RIPA lysis buffer, the reagent of Coomassie Plus^TM^ Protein Assay, and the enhanced chemiluminescence (ECL) reagent were purchased from Thermo Fisher Scientific (Rockford, IL, USA). The apoptosis assay kit was purchased from Bio-Legend (San Diego, CA, USA). The sulforhodamine B (SRB) reagent was obtained from Sigma-Aldrich, St. Louis, MO, USA. The propidium iodide (PI) dye, and primary antibody for anti-β-actin were purchased from Sigma-Aldrich (St. Louis, MO, USA). The primary antibodies for Western blot analysis (cyclin D1, cyclin E1, CDK-2, CDK-4, caspase-9, cleaved-caspase-3, Bcl-2, Bcl-xl, and HRP-conjugated anti-mouse or rabbit-IgG) were obtained from Cell Signaling Technology (Beverly, MA, USA). The primary antibody of PARP-1 was purchased from Santa Cruz Biotechnology (Dallas, TX, USA). The MitoView^TM^ 633 dye staining kit was obtained from Biotium (Fremont, CA, USA).

### 4.3. Preparation of Herbal Extracts

In the process of preparing herbal extracts, the herbal materials, namely *Dracaena loureiri, Harrisonia perforata*, and *Ficus racemose*, were carefully dried in a shaded environment and subsequently stored. Each of the dried herbs, approximately weighing 100 g, was mixed with 4 L of 80% (*v*/*v*) ethanol solution, and this mixture was allowed to steep for a duration of 48 h to facilitate the extraction process. Following the steeping period, the mixture underwent a filtration step to remove solid residues, resulting in a filtrate containing the extracted components. This filtrate, termed the “extract” was then concentrated using a rotary vacuum evaporator (BUCHI, Switzerland) to obtain ethanolic fractions. These concentrated ethanolic fractions were then subjected to freeze-drying, resulting in the production of powdered ethanolic extracts, which were identified as DLEE (*Dracaena loureirin* ethanolic extract), FREE (*Ficus racemose* ethanolic extract), and HPEE (*Harrisonia perforate* ethanolic extract). To preserve the stability and integrity of the extracts, they were stored at −20 °C for further experimentation.

### 4.4. Total Phenolic Content

The total phenolic content of three ethanolic extracts was determined by the modified Folin-Ciocalteu assay as described in the previous study [50]. Briefly, each ethanolic extracts concentration (400 µL) was mixed with 300 µL of Folin-Ciocalteau reagent for 3 min at the room temperature (dark). Following this, 300 µL of 7.5% (*w*/*v*) sodium carbonate (Na_2_CO_3_) was added to the mixture, and then incubation more for 30 min. The mixture was measured at 765 nm using a UV-visible spectrophotometer (UV-1800, Shimadzu Co., Ltd., Kyoto, Japan). The standard curve was prepared with various concentrations of gallic acid (GA) solution. The data were expressed as milligrams of GA equivalents per gram of the ethanolic extracts (mg GA/g extract).

### 4.5. Total Flavonoid Content

The total flavonoid contents of three ethanolic extracts were determined by the aluminum chloride (AlCl_3_) colorimetric assay as described in the previous study [51]. Each ethanolic extracts concentration (250 µL) was mixed with 125 µL of 5% (*w*/*v*) NaNO_2_ for 5 min at room temperature (dark). Next, 125 µL of 10% (*w*/*v*) AlCl_3_ was added to the mixture and incubated more for 5 min. Then, 1000 µL of 1N NaOH was added and incubated for 15 min at room temperature. The mixture was measured at 510 nm using a UV–visible spectrophotometer (UV-1800, Shimadzu Co., Ltd., Kyoto, Japan). The standard curve was prepared with various concentrations of catechin (CE) solution. The total flavonoid content was expressed as milligrams of CE equivalent per gram of ethanolic extract (mg CE/g extract).

### 4.6. Phytochemicals Content Determination Using HPLC

The HPLC analysis of DLEE was performed on a HPLC (Agilent Tecnologies, Santa Clara, CA, USA) using a reversed-phase C18 (250 mm × 4.6 mm, 5 µm) column. The flavonoid compounds (resveratrol, rutin, quercetin, quercitrin, catechin, luteolin, apigenin, hesperetin, and hesperidin) were analyzed using gradient conditions [52]. The mobile phase composition was established with 0.2% formic acid in water (mobile phase A) and 100% methanol (mobile phase B), initially set at 70% A: 30% B at 0 min. The gradient elution was programmed as follows: 0–5 min, transitioning from 70% to 65% A; 5.01–40 min, transitioning from 65% to 60%; 40.01–50 min, transitioning from 60% to 55%; 50.01–75 min, transitioning from 55% to 54%; 75.01–80 min, transitioning from 54% to 40%; maintaining a stable 40% A from 80.01 to 85 min and then shifting from 40% to 70% A between 85.01 and 90 min. Detection wavelengths were set at 280 and 330 nm, and the samples were processed for 90 min at a flow rate of 1.0 mL/min. The loureirin A and loureirin B were analyzed using the isocratic conditions [53]. The mobile phase composition was established with 0.1% acetic acid in water (mobile phase A) and 100% acetonitrile (mobile phase B), the mobile phase was set at 63% A: 37% B during the experiment. Detection wavelengths were set at 280, and the samples were processed for 60 min at a flow rate of 1.0 mL/min. The quantitative data of the phytochemical compounds in DLEE were calculated from their respective calibration curves from standard loureirin A, loureirin B, resveratrol, rutin, quercetin, quercitrin, catechin, luteolin, apigenin, hesperetin, and hesperidin to obtain the concentrations of all the compounds (mg/g of extract).

### 4.7. Cell Line and Cell Culture

A549 cells, which are a non-small-cell lung carcinoma (NSCLC) phenotype and primary dermal fibroblast (human), which is a control for normal healthy cells, were purchased from ATCC (Manassas, VA, USA). Both cell lines were cultured in DMEM with 10% FBS, 50 IU/mL penicillin, and 50 g/mL streptomycin. The cells were maintained in a 5% CO_2_ humidified incubator at 37 °C. The harvesting and seeding the cells for subsequent experiments were performed when the cells reached 70–80% confluency.

### 4.8. Cell Viability Assay

The cytotoxicity of ethanolic extracts against A549 and human normal fibroblast cells were determined by a sulforhodamine B (SRB) assay as described in the previous study [54]. The A549 cells (5 × 10^3^ cells/well) or human normal fibroblast cells (4 × 10^3^ cells/well) were plated in a 96-well plate and incubated overnight. Next, the cells were treated with or without ethanolic extracts at various concentrations (0–200 μg/mL) and incubated for 24 and 48 h. Following incubation, 100 µL 10% (*w*/*v*) trichloroacetic acid was introduced to the cells, and the cells were then kept at 4 °C for 1 h. Subsequently, the medium was aspirated, and the cells were rinsed using slow-running tap water. After drying, the cells were stained with 100 μL of 0.054% (*w*/*v*) SRB solution and incubated for 30 min at room temperature. For removing the SRB solution, the cells were washed with 1% (*v*/*v*) acetic acid 4 times. The dye was dissolved with 150 μL of 10 mM tris-based solution (pH 10.5) and the absorbance was measured at 510 nm using a microplate reader (Sunrise, Tecan Trading AG, Männedorf, Switzerland). The cell viability was calculated compared to the control.

### 4.9. Colony Formation Assay

The effect of DLEE on diminishing A549 cell proliferation was examined by a colony formation assay, following the established methodology detailed in the previous protocol [55]. The A549 cells (500 cells/well) were plated in a 6-well plate and incubated overnight. The cells were treated with various concentrations of DLEE (0–60 μg/mL) for 7 days before being fixed. The cells were fixed using 6% glutaraldehyde and incubated for 30 min. Subsequently, the cells were stained with Toluidine dye and incubated for 45 min. The dye was then removed by slow-running tap water. The images were captured by the iBright™ CL-1500 imaging system (Thermo Fisher Scientific). Colony numbers were quantified using ImageJ 1.410 software https://imagej.nih.gov/ij/ (accessed on 15 September 2023). Each experiment involved triplicate determinations.

### 4.10. Cell Cycle Assay

The A549 cells (1 × 10^6^ cells/well) were plated in a 6-well plate and incubated overnight. Next, the cells were starved with 0.5% FBS in DMEM for 18 h. The A549 cells were treated with DLEE in various concentrations (0–60 μg/mL) and incubated for 24 h. After incubation, the cells were harvested and were fixed with 70% methanol overnight at −20 °C. Then, the cells were stained with PI solution (20 mg/mL) and incubated for 45 min at room temperature. The amount of cellular DNA content in the samples was analyzed using a flow cytometer (Beckman Coulter Inc., Indianapolis, IN, USA). The cell population in each phase of the cell cycle was quantified using CytExpert 2.0 software for the CytoFLEX platform (Beckman Coulter Inc., Indianapolis, IN, USA).

### 4.11. Apoptosis Assay

The percentage of apoptotic cells was assessed utilizing an Annexin V-FITC/PI apoptosis detection kit (Bio-Legend, San Diego, CA, USA). The A549 cells (1 × 10^6^ cells/well) were plated in a 6-well plate and incubated overnight. Then, the cells were treated with various concentrations of DLEE for 48 h before being harvested. The cells were harvested and then washed with PBS 2 times. Followed by staining with 5 µL of Annexin V-FITC and 10 µL of PI dye. Then, the cells were incubated at room temperature (dark) for 15 min. After that, 400 µL of reagent buffer was added to the mixture. The apoptotic cells were detected using a flow cytometer (Beckman Coulter Inc., Indianapolis, IN, USA) within 30 min.

### 4.12. Mitochondrial Membrane Potential

The mitochondrial membrane potential (MMP) was evaluated using a MitoView^TM^ 633 dye staining kit obtained from Biotium, Fremont, CA, USA. According to the manufacturer’s protocol, the A549 cells (1 × 10^6^ cells/well) were plated in a 6-well plate and incubated overnight. Then, the cells were treated with various concentrations of DLEE for 24 h before being harvested. The cells were incubated with 100 nM of MitoView^TM^ 633 dye in incomplete DMEM for 20 min in a 5% CO_2_ incubator. After incubation, the cells were washed with PBS and were analyzed using a flow cytometer (Beckman Coulter Inc., Indianapolis, IN, USA) at excitation/emission of 638/660 nm. The data were analyzed using CytExpert for DxFLEX 2.0 software.

### 4.13. Western Blot Analysis

The A549 cells were lysed using RIPA lysis buffer (100 μL/well) and centrifuged at 10,000 rpm for 10 min at 4 °C. The Bradford assay was used to measure the concentration of the protein samples. Equal quantities of protein samples (20 μg) were loaded on a 12% SDS-gel, resolved using SDS-PAGE, and transferred to 0.45 μm PVDF/nitrocellulose membranes which were soaked in methanol for 10 min in advance. Next, the membranes were blocked with 5% BSA in 0.05% TBS-Tween (TBS-T) for 1 h at room temperature, washed 3 times with 0.05% TBS-T, and incubated with primary antibodies against cyclin D1, cyclin E1, CDK-4, CDK-2, cleaved-caspase3, Bcl-2, Bcl-xl, survivin, and PARP-1 overnight at 4 °C. Next, after washing the membranes 4 times with 0.5% TBS-T, the membranes were incubated with goat anti-mouse or rabbit HRP-conjugated secondary antibody for 2 h at room temperature and were then washed 4 times with 0.05% TBS-T. The protein bands were visualized using a chemiluminescence imaging system following treatment with HRP substrate (ECL) for 1–4 min. β-actin was used as the loading control. Band density levels were analyzed using Image J 1.410.

### 4.14. Statistical Analysis

Statistical analysis was performed using Prism version 8.0 software based on three independent experiments. All experiments were conducted in triplicate independent trials to ensure reproducibility. The independent *t*-test and one-way ANOVA with Dunnett’s test were employed for data analysis across various experiments, including total phenolic and total flavonoid content cell viability assay, colony formation assay, cell cycle assay, apoptosis assay, mitochondrial membrane potential assay, as well as Western blot assay data. The data were reported as mean ± standard deviation (mean ± S.D.). Statistical significance was established at * *p* < 0.05, ** *p* < 0.01, and *** *p* < 0.001.

## Figures and Tables

**Table 1:** Total phenolic and total flavonoid contents of DLEE, HPEE, and FREE.

Herb Extracts	Total Phenolic Content (mg of GAE/g of Extract)	Total Flavonoid Content (mg of CE/g of Extract)
DLEE	251.49 ± 23.13 ***	85.83 ± 4.80 ***
HPEE	193.53 ± 3.21	62.13 ± 1.22
FREE	89.05 ± 3.42	46.64 ± 1.59

*** *p* < 0.001 vs. other herb extracts using an independent *t*-test. The data are displayed as mean ± S.D. values from three independent experiments.

**Table 2:** Identification of phytochemical compounds in DLEE using the HPLC technique.

Standard Compounds	mg/g of Extract (Mean ± S.D.)
Loureirin A	28.11 ± 0.34
Quercetin	25.81 ± 1.12
Quercitrin	6.48 ± 0.51
Loureirin B	6.27 ± 0.15
Hesperetin	6.14 ± 0.18
Rutin	5.86 ± 0.27
Catechin	3.32 ± 0.46
Resveratrol	2.65 ± 0.03
Hesperidin	1.98 ± 0.26
Luteolin	ND
Apigenin	ND

ND = Not detectable. The data are displayed as mean ± S.D. values from three independent experiments.

## Data Availability

Data are contained within the article.

## References

[1] Sung H., Ferlay J., Siegel R.L., Laversanne M., Soerjomataram I., Jemal A., Bray F. (2021). Global cancer statistics 2020: GLOBOCAN estimates of incidence and mortality worldwide for 36 cancers in 185 countries. CA A Cancer J. Clin..

[2] Chen P., Liu Y., Wen Y., Zhou C. (2022). Non-small cell lung cancer in China. Cancer Commun..

[3] Huang C.-Y., Ju D.-T., Chang C.-F., Reddy P.M., Velmurugan B.K. (2017). A review on the effects of current chemotherapy drugs and natural agents in treating non–small cell lung cancer. Biomedicine.

[4] Kumar M., Sarkar A. (2022). Current therapeutic strategies and challenges in NSCLC treatment: A comprehensive review. Exp. Oncol..

[5] Fennell D., Summers Y., Cadranel J., Benepal T., Christoph D., Lal R., Das M., Maxwell F., Visseren-Grul C., Ferry D. (2016). Cisplatin in the modern era: The backbone of first-line chemotherapy for non-small cell lung cancer. Cancer Treat. Rev..

[6] Group I.A.L.C.T.C. (2004). Cisplatin-based adjuvant chemotherapy in patients with completely resected non–small-cell lung cancer. N. Engl. J. Med..

[7] Cragg G.M., Grothaus P.G., Newman D.J. (2009). Impact of natural products on developing new anti-cancer agents. Chem. Rev..

[8] Wu J., Li Y., He Q., Yang X. (2023). Exploration of the Use of Natural Compounds in Combination with Chemotherapy Drugs for Tumor Treatment. Molecules.

[9] Sofi M.S., Nabi S., Mohammed C., Sofi S. (2018). The role of phytocompounds in cancer treatment: A current review. J. Med. Plant Stud..

[10] Sukhramani P.S., Vidyasagar G., Patel P.M. (2013). In-vitro screening of Ficus racemosa for Anticancer activity. Res. J. Pharmacogn. Phytochem..

[11] Dharmadeva S., Galgamuwa L.S., Prasadinie C., Kumarasinghe N. (2018). In vitro anti-inflammatory activity of *Ficus racemosa* L.. bark using albumin denaturation method. Ayu.

[12] Ahmed F., Urooj A. (2010). Traditional uses, medicinal properties, and phytopharmacology of Ficus racemosa: A review. Pharm. Biol..

[13] Juckmeta T., Pipatrattanaseree W., Jaidee W., Dechayont B., Chunthorng-Orn J., Andersen R.J., Itharat A. (2019). Cytotoxicity to five cancer cell lines of the respiratory tract system and anti-inflammatory activity of Thai traditional remedy. Nat. Prod. Commun..

[14] Nguyen-Pouplin J., Tran H., Tran H., Phan T.A., Dolecek C., Farrar J., Tran T.H., Caron P., Bodo B., Grellier P. (2007). Antimalarial and cytotoxic activities of ethnopharmacologically selected medicinal plants from South Vietnam. J. Ethnopharmacol..

[15] Somsil P., Ruangrungsi N., Limpanasitikul W., Itthipanichpong C. (2012). In vivo and in vitro anti-inflammatory activity of Harrisonia perforata root extract. Pharmacogn. J..

[16] Choodej S., Sommit D., Pudhom K. (2013). Rearranged limonoids and chromones from Harrisonia perforata and their anti-inflammatory activity. Bioorg. Med. Chem. Lett..

[17] Cheenpracha S., Chokchaisiri R., Ganranoo L., Maneerat T., Rujanapun N., Charoensup R., Laphookhieo S., Injan N., Nokbin S. (2022). Isoprenylated chromones from the stems of Harrisonia perforata. Phytochem. Lett..

[18] Chea A., Jonville M.-C., Bun S.-S., Laget M., Elias R., Duménil G., Balansard G. (2007). In vitro antimicrobial activity of plants used in Cambodian traditional medicine. Am. J. Chin. Med..

[19] Thu Z.M., Myo K.K., Aung H.T., Armijos C., Vidari G. (2020). Flavonoids and stilbenoids of the genera Dracaena and Sansevieria: Structures and bioactivities. Molecules.

[20] Ichikawa K., Kitaoka M., Taki M., Takaishi S., Boriboon M., Akiyama T. (1997). Retrodihydrochalcones and homoisoflavones isolated from Thai medicinal plant Dracaena loureiri and their estrogen agonist activity. Planta Med..

[21] Reanmongkol W., Subhadhirasakul S., Bouking P. (2003). Antinociceptive and antipyretic activities of extracts and fractions from Dracaena loureiri in experimental animals. Songklanakarin J. Sci. Technol..

[22] Sun J., Liu J.-N., Fan B., Chen X.-N., Pang D.-R., Zheng J., Zhang Q., Zhao Y.-F., Xiao W., Tu P.-F. (2019). Phenolic constituents, pharmacological activities, quality control, and metabolism of Dracaena species: A review. J. Ethnopharmacol..

[23] Sun X., Wen K., Xu Z., He Z., Wu B., Yang X., Wang X. (2020). Effect of Loureirin B on Crohn’s disease rat model induced by TNBS via IL-6/STAT3/NF-κB signaling pathway. Chin. Med..

[24] Chen M.J., Cheng A.C., Lee M.F., Hsu Y.C. (2018). Simvastatin induces G1 arrest by up-regulating GSK3β and down-regulating CDK4/cyclin D1 and CDK2/cyclin E1 in human primary colorectal cancer cells. J. Cell. Physiol..

[25] Jamshidi-Kia F., Lorigooini Z., Amini-Khoei H. (2017). Medicinal plants: Past history and future perspective. J. Herbmed Pharmacol..

[26] Gurib-Fakim A. (2006). Medicinal plants: Traditions of yesterday and drugs of tomorrow. Mol. Asp. Med..

[27] Desai A.G., Qazi G.N., Ganju R.K., El-Tamer M., Singh J., Saxena A.K., Bedi Y.S., Taneja S.C., Bhat H.K. (2008). Medicinal plants and cancer chemoprevention. Curr. Drug Metab..

[28] Khan M.I., Bouyahya A., Hachlafi N.E., Menyiy N.E., Akram M., Sultana S., Zengin G., Ponomareva L., Shariati M.A., Ojo O.A. (2022). Anticancer properties of medicinal plants and their bioactive compounds against breast cancer: A review on recent investigations. Environ. Sci. Pollut. Res..

[29] Jain R., Venkatasubramanian P. (2014). Proposed correlation of modern processing principles for Ayurvedic herbal drug manufacturing: A systematic review. Anc. Sci. Life.

[30] Sánchez-Martínez C., Lallena M.J., Sanfeliciano S.G., de Dios A. (2019). Cyclin dependent kinase (CDK) inhibitors as anticancer drugs: Recent advances (2015–2019). Bioorg. Med. Chem. Lett..

[31] Maddika S., Ande S.R., Panigrahi S., Paranjothy T., Weglarczyk K., Zuse A., Eshraghi M., Manda K.D., Wiechec E., Los M. (2007). Cell survival, cell death and cell cycle pathways are interconnected: Implications for cancer therapy. Drug Resist. Updates.

[32] Evan G.I., Brown L., Whyte M., Harrington E. (1995). Apoptosis and the cell cycle. Curr. Opin. Cell Biol..

[33] Vaux D.L., Korsmeyer S.J. (1999). Cell death in development. Cell.

[34] Ly J.D., Grubb D.R., Lawen A. (2003). The mitochondrial membrane potential (Δψ m) in apoptosis; an update. Apoptosis.

[35] Gottlieb E., Armour S., Harris M., Thompson C. (2003). Mitochondrial membrane potential regulates matrix configuration and cytochrome c release during apoptosis. Cell Death Differ..

[36] Niu S., Liu T., Deng Y., Wang W., Zhang Y., Hong W., Zhang D., Hua J., Luo S. (2020). Production and evaluation of antifungal stilbenoids in Dracaena cochinchinensis elicited by fungal inoculation. Ind. Crops Prod..

[37] Ouncharoen K., Itharat A., Chaiyawatthanananthn P. (2017). In vitro free radical scavenging and cell-based antioxidant activities of Kheaw-Hom remedy extracts and its plant ingredients. J. Med. Assoc. Thail..

[38] Dechayont B., Phuaklee P., Chunthorng-Orn J., Juckmeta T., Prajuabjinda O., Jiraratsatit K. (2021). Antibacterial, anti-inflammatory and antioxidant activities of Mahanintangtong and its constituent herbs, a formula used in Thai traditional medicine for treating pharyngitis. BMC Complement. Med. Ther..

[39] Likhitwitayawuid K., Sawasdee K., Kirtikara K. (2002). Flavonoids and stilbenoids with COX-1 and COX-2 inhibitory activity from Dracaena loureiri. Planta Med..

[40] Meksuriyen D., Cordell G.A. (1988). Traditional medicinal plants of Thailand XIII. Flavonoid derivatives from Dracaena loureiri (Agavaceae). Sci. Asia.

[41] Hao Q., Saito Y., Matsuo Y., Li H.-Z., Tanaka T. (2015). Chalcane–stilbene conjugates and oligomeric flavonoids from Chinese dragon’s blood produced from Dracaena cochinchinensis. Phytochemistry.

[42] Hu S.-L., Wang K., Shi Y.-F., Shao Z.-X., Zhang C.-X., Sheng K.-W., Ge Z.-D., Chen J.-X., Wang X.-Y. (2020). Downregulating Akt/NF-κB signaling and its antioxidant activity with Loureirin A for alleviating the progression of osteoarthritis: In vitro and vivo studies. Int. Immunopharmacol..

[43] Zheng S.-Y., Li Y., Jiang D., Zhao J., Ge J.-F. (2012). Anticancer effect and apoptosis induction by quercetin in the human lung cancer cell line A-549. Mol. Med. Rep..

[44] Yuan L., Zhang Y., Xia J., Liu B., Zhang Q., Liu J., Luo L., Peng Z., Song Z., Zhu R. (2015). Resveratrol induces cell cycle arrest via a p53-independent pathway in A549 cells. Mol. Med. Rep..

[45] Takashina M., Inoue S., Tomihara K., Tomita K., Hattori K., Zhao Q.-L., Suzuki T., Noguchi M., Ohashi W., Hattori Y. (2017). Different effect of resveratrol to induction of apoptosis depending on the type of human cancer cells. Int. J. Oncol..

[46] Gosslau A., Pabbaraja S., Knapp S., Chen K.Y. (2008). Trans-and cis-stilbene polyphenols induced rapid perinuclear mitochondrial clustering and p53-independent apoptosis in cancer cells but not normal cells. Eur. J. Pharmacol..

[47] Wu F., Chen J., Fan L.M., Liu K., Zhang N., Li S.W., Zhu H., Gao H.C. (2017). Analysis of the effect of rutin on GSK-3β and TNF-α expression in lung cancer. Exp. Ther. Med..

[48] Birsu Cincin Z., Unlu M., Kiran B., Sinem Bireller E., Baran Y., Cakmakoglu B. (2015). Anti-proliferative, apoptotic and signal transduction effects of hesperidin in non-small cell lung cancer cells. Cell. Oncol..

[49] Xia R., Sheng X., Xu X., Yu C., Lu H. (2018). Hesperidin induces apoptosis and G0/G1 arrest in human non-small cell lung cancer A549 cells. Int. J. Mol. Med..

[50] Noreen H., Semmar N., Farman M., McCullagh J.S. (2017). Measurement of total phenolic content and antioxidant activity of aerial parts of medicinal plant Coronopus didymus. Asian Pac. J. Trop. Med..

[51] Shraim A.M., Ahmed T.A., Rahman M.M., Hijji Y.M. (2021). Determination of total flavonoid content by aluminum chloride assay: A critical evaluation. LWT.

[52] Wu J., Xing H., Tang D., Gao Y., Yin X., Du Q., Jiang X., Yang D. (2012). Simultaneous determination of nine flavonoids in beagle dog by HPLC with DAD and application of Ginkgo biloba extracts on the pharmacokinetic. Acta Chromatogr..

[53] Hu X., Wang X., Liu M., Tai Z., Li G. (2014). Quantitative evaluation of loureirin A and loureirin B in Dragon’s blood capsules from different manufacturers by HPLC. J. Chem. Pharm. Res..

[54] Orellana E.A., Kasinski A.L. (2016). Sulforhodamine B (SRB) assay in cell culture to investigate cell proliferation. Bio Protoc..

[55] Franken N.A., Rodermond H.M., Stap J., Haveman J., Van Bree C. (2006). Clonogenic assay of cells in vitro. Nat. Protoc..

